# TRPC channels in exercise-mimetic therapy

**DOI:** 10.1007/s00424-018-2211-3

**Published:** 2018-10-08

**Authors:** Takuro Numaga-Tomita, Sayaka Oda, Kazuhiro Nishiyama, Tomohiro Tanaka, Akiyuki Nishimura, Motohiro Nishida

**Affiliations:** 1 Division of Cardiocirculatory Signaling, National Institute for Physiological Sciences (NIPS), National Institutes of Natural Sciences, Higashiyama 5-1, Myodaiji-cho, Okazaki, Aichi 444-8787 Japan; 2 Cardiocirculatory Dynamism Research Group, Exploratory Research Center on Life and Living Systems (ExCELLS), National Institutes of Natural Sciences, Okazaki, Aichi 444-8787 Japan; 3 SOKENDAI (School of Life Science, The Graduate University for Advanced Studies), Hayama, Kanagawa Japan; 4 Department of Translational Pharmaceutical Sciences, Graduate School of Pharmaceutical Sciences, Kyushu University, Fukuoka, 812-8582 Japan

**Keywords:** Transient receptor potential, Redox signaling, Remodeling, Plasticity, Protein-protein interaction

## Abstract

Physical exercise yields beneficial effects on all types of muscle cells, which are essential for the maintenance of cardiovascular homeostasis and good blood circulation. Daily moderate exercise increases systemic antioxidative capacity, which can lead to the prevention of the onset and progression of oxidative stress-related diseases. Therefore, exercise is now widely accepted as one of the best therapeutic strategies for the treatment of ischemic (hypoxic) diseases. Canonical transient receptor potential (TRPC) proteins are non-selective cation channels activated by mechanical stress and/or stimulation of phospholipase C-coupled surface receptors. TRPC channels, especially diacylglycerol-activated TRPC channels (TRPC3 and TRPC6; TRPC3/6), play a key role in the development of cardiovascular remodeling. We have recently found that physical interaction between TRPC3 and NADPH oxidase (Nox) 2 under hypoxic stress promotes Nox2-dependent reactive oxygen species (ROS) production and mediates rodent cardiac plasticity, and inhibition of the TRPC3-Nox2 protein complex results in enhancement of myocardial compliance and flexibility similar to that observed in exercise-treated hearts. In this review, we describe current understanding of the roles of TRPC channels in striated muscle (patho)physiology and propose that targeting TRPC-based protein complexes could be a new strategy to imitate exercise therapy.

## Introduction

Moderate exercise produces pleiotropic effects on health, by stimulating systemic blood circulation and energy metabolism and refreshing the mind. Exercise is also known to reduce cardiovascular risk factors related to many pathological conditions. Although primarily affecting skeletal muscle functions such as increasing the rate of glucose uptake, physical exercise gives rise to several beneficial effects on other remote organs through blood circulation-dependent organ-organ interactions. A critical risk factor for the development of pathological conditions is the increase of oxidative stress caused by overproduction of reactive oxygen species (ROS). ROS are mainly produced as a byproduct of uncoupling of the respiratory chain during mitochondrial dysfunction, or enzymatically, by nicotinamide adenine dinucleotide phosphate (NADPH) oxidase (Nox) proteins. Physical exercise is well known to induce ROS production, but it also triggers upregulation of antioxidative systems leading to acquisition of resilience against many diseases [65]. We have also revealed that long-term physical exercise reduces the expression of Nox2 in the heart [69]. This review introduces the (patho)physiological roles of Nox and canonical transient receptor potential (TRPC) proteins in skeletal muscles, mainly focusing on the mechanisms common to other muscular organs (i.e., the cardiovascular system), and proposes a novel strategy for imitating exercise therapy.

## Roles of Nox proteins in skeletal muscle

There are seven isoforms of Nox proteins. Nox1, Nox2 and Nox4 are reportedly expressed in skeletal muscle [15]. Nox2 and Nox4 are predominantly expressed in the heart. In resting conditions, Nox2 only interacts with the p22^phox^ subunit of the NADPH oxidase, which promotes the expression of Nox2 by preventing its proteasomal degradation. Upon cellular activation, the other cytoplasmic subunits p67^phox^, p40^phox^ and p47^phox^, and the small G protein Rac1 are recruited and activate Nox2 protein. Among the cytoplasmic subunits, p47^phox^ is the main regulator of the Nox2 complex formation. To form a complex, phosphorylation of p47^phox^ is necessary. Phosphorylation of p47^phox^ is reported to be mediated by protein kinase C, mitogen-activated protein kinases and p21-activated kinase [13]. The importance of Nox proteins in skeletal muscle is highlighted by their role in contraction-induced ROS production [25]. It is well known that muscle contraction produces ROS and reactive nitrogen species [26, 59]. ROS production plays important roles in skeletal muscle, for example, increasing the activities of antioxidant defense enzymes, force production, glucose uptake and insulin signaling [25, 45]. Application of hydrogen peroxide (H_2_O_2_) induces a similar gene expression profile to that of contracting a skeletal muscle, suggesting that muscle contraction signals are mainly conveyed by H_2_O_2_ [46]. The regulation and physiological relevance of Nox proteins in skeletal muscle have been reviewed in detail elsewhere [15, 27].

## Roles of TRPC channels in skeletal muscle

The *trp* gene was first identified in 1989 as a causative gene mutant affecting phototransduction in *Drosophila* [49]. Twenty-eight mammalian TRP homologues have been identified, and these are subdivided into six subfamilies based on their genetic and functional similarities: TRPC (canonical), TRPV (vanilloid), TRPM (melastatin), TRPP (polycystin), TRPML (mucolipin) and TRPA (ankyrin). TRP proteins commonly possess six transmembrane domains and a preserved 25-amino acid sequence called the ‘TRP domain’. There are several reports demonstrating the involvement of TRP channels in exercised skeletal muscles. TRPM8 activation enhances exercise endurance and reduces blood lactic acid and triglycerides by upregulating uncoupling protein 1 (UCP1) and peroxisome proliferator-activated receptor-γ coactivator 1α (PGC1α) in skeletal muscles [36]. TRPV1 activation by dietary capsaicin increases the proportion of oxidative fibers, promotes mitochondrial biogenesis, enhances exercise endurance and prevents high-fat diet-induced metabolic disorders via an increase of PGC1α expression [41]. TRPV1 is reportedly activated by peroxynitrite, a reaction product of nitric oxide and superoxide, and mediates overload-induced skeletal muscle hypertrophy [23, 24]. These TRP channels are likely to function downstream of mechano-signal transduction in skeletal muscle contraction.

**Fig. 1:**
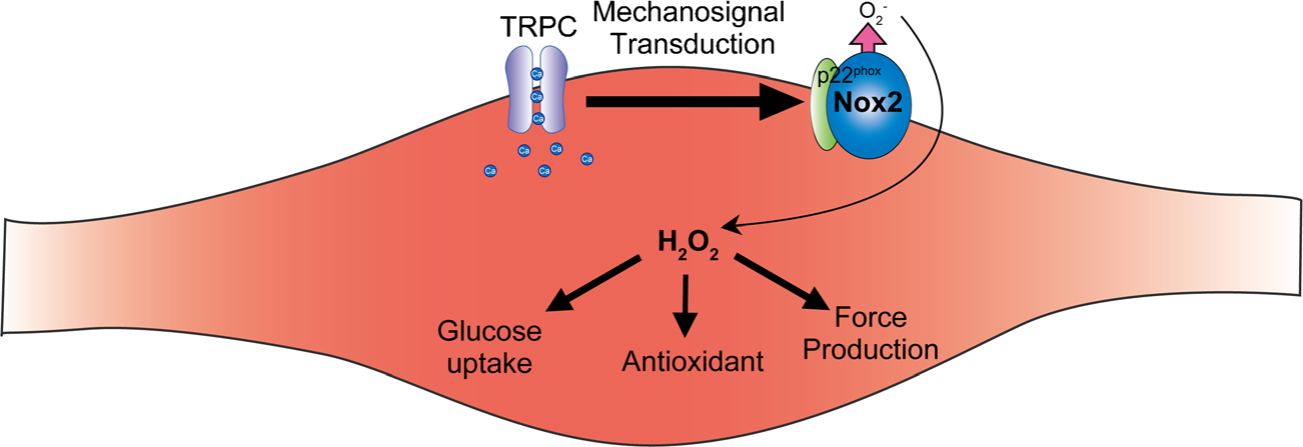
Canonical transient receptor potential (TRPC) channels function as mechanosignal transducers to Nox proteins during skeletal muscle contraction. Nox-mediated reactive oxygen species (ROS) production plays critical roles in skeletal muscle homeostasis

The TRPC family proteins, comprising seven mammalian homologues (TRPC1–TRPC7), are believed to be molecular candidates for receptor-activated cation channels (RACCs) [49]. TRPC1 was first suggested as the molecular entity of store-operated Ca^2+^ entry (SOCE) [38, 78, 95, 96]. TRPC1 contributes to the coordination of elementary Ca^2+^ signaling events through promoting functional coupling between the endoplasmic reticulum (ER) and the plasma membrane in receptor-induced Ca^2+^ signaling [50]. Recent findings indicate that TRPC proteins have two important roles: one is to act as a critical component of stretch-activated or store-operated Ca^2+^-permeable channels, and the other is to act as a signaling platform to amplify receptor-activated Ca^2+^ signaling via interacting with intracellular signaling molecules [52, 54]. Because of their universal activation mechanism in many cell types, TRPC channels play important roles in basic cellular responses including proliferation, differentiation and death in response to various environmental stimuli. TRPC channels are also linked to physical stimulation such as mechanical stretch, and hypoxia and oxidative stress [62]. TRPC1 and TRPC6 are suggested to be components of the tarantula toxin-sensitive mechanosensitive cation channel [42, 70]. In addition, intracellular lipid mediators such as diacylglycerol and 20-hydroxyeicosatetraenoic acid (20-HETE) mediate activation of TRPC6 induced by oxidative stress [77] and mechanical stretch [22]. Considering the role of TRPC3/6 heterotetramer channels in myocyte hypertrophy, the TRPC6 protein signaling complex, including TRPC1 and TRPC3, may function as a mechanical signal transducer in striated muscle cells (Fig. 1).

## TRPC1

**Fig. 2:**
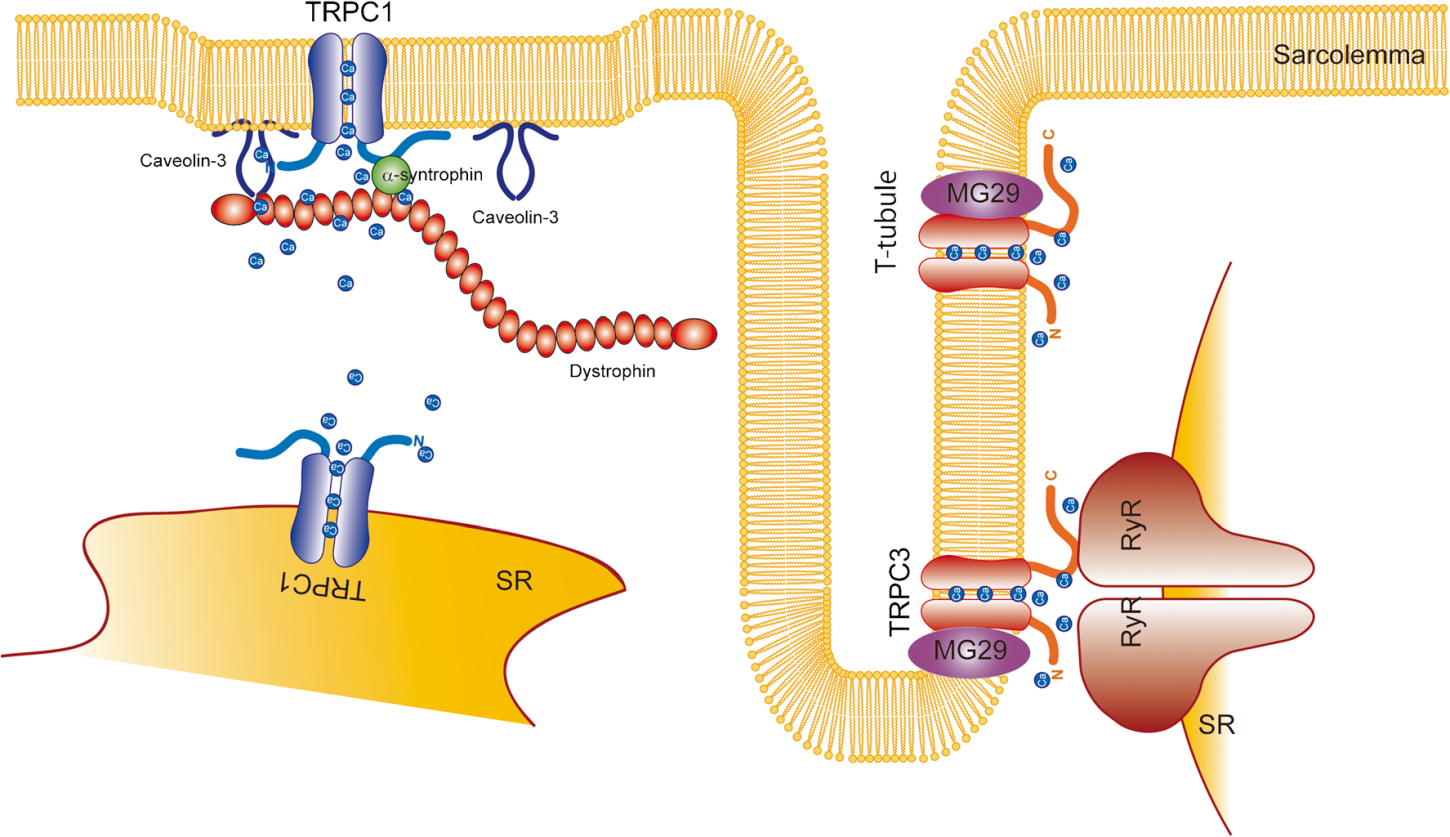
Canonical transient receptor potential (TRPC) channel function in striated muscle cells. TRPC1 channel activity is regulated via interaction with the dystrophin-associated protein complex (DAPC). TRPC1 also functions as a Ca^2+^ leak channel in the sarcoplasmic reticulum. TRPC3 channels are localized in T-tubules

Vandebrouck et al. first demonstrated that TRPC1/2/3/4 and TRPC6 were detected both at the transcript and protein levels in skeletal muscle cells, with TRPC2 and TRPC3 being found in intracellular compartments, and TRPC1/4 and TRPC6 at the plasma membrane [75]. The abnormal Ca^2+^ influx observed in adult skeletal muscle fibers from dystrophic (mdx) mice was partially mediated by TRPC channels [75]. Later, the same group demonstrated that TRPC1 is associated with the PSD95-discs large-zonula occludens protein (PDZ) domain-possessing scaffold proteins α1-syntrophin and dystrophin and suggested that the mechanosensitive activation of TRPC1 is supported by these interactions (Fig. 1) [74]. Stiber et al. demonstrated that Homer1 determines the localization and activation timing by mechanical stretch of TRPC1 channels. Therefore, the absence of Homer1 induces spontaneous TRPC1 activation and Ca^2+^ overload which results in myopathy [71]. Another group demonstrated that protein levels of TRPC1 and Caveolin-3 (Cav3) were increased in skeletal muscle from mdx mice and that TRPC1 was activated by ROS in an Src kinase-dependent manner (Fig. 2) [18].

TRPC1 mediates SOCE in the C2C12 myoblast cell line. siRNA-mediated knockdown of TRPC1 suppressed myotube formation of C2C12 cells. Interestingly, TRPC1 mRNA expression transiently increased immediately after the onset of differentiation (1 day) and returned to the basal level 4–6 days after the start of differentiation. Increased TRPC1 activity was correlated with the activity of calpain [40]. TRPC1 proteins were also transiently upregulated 24 h after the induction of differentiation and returned to the basal level at 72 h. Formigli et al. also demonstrated that TRPC1 is not only activated by store depletion, but also mechanical stretch, in C2C12 cells. Mechanical stretch facilitates myoblast differentiation in a sphingosine 1-phosphate (S1P)-dependent manner [12]. S1P application to C2C12 cells markedly increased TRPC1 expression, concomitant with an increase in stretch-activated channel expression [17]. S1P-mediated activation of TRPC1 induces m-calpain activity and subsequent expression of connexin43 [47]. TRPC1 overexpression in C2C12 cells increased the rate and amplitude of SOCE. Interestingly, in those cells levels of stromal interaction molecule 1 (STIM1) and sarcoendoplasmic reticulum calcium ATPase (SERCA) expression were lower, which resulted in insufficient Ca^2+^ clearance after the depolarization-induced Ca^2+^ increase. Furthermore, Ca^2+^ dyshomeostasis induced by TRPC1 overexpression attenuated the nuclear factor of activated T cells (NFAT) signaling pathway and myotube formation [57]. In human myoblasts, TRPC1 downregulation caused by siRNA expression or overexpression of a dominant negative mutant clearly suppressed SOCE, myogenic driver MEF2 expression and fusion of myoblasts into myotubes [3]. TRPC1 activation is regulated by STIM1L, a long isoform of STIM1 [2]. TRPC1 forms a heterotetramer with TRPC3 via interaction at the ankyrin repeat of TRPC3. The short protein comprising the N-terminal 37 amino acids of TRPC3 can inhibit TRPC1-TRPC3 heteromultimerization, which reduces resting cytosolic Ca^2+^ in murine skeletal myotubes [82].

TRPC1 is highly expressed in skeletal muscle stem cell satellite cells. Fibroblast growth factor 2 (FGF2) treatment increased the intracellular Ca^2+^ concentration and nuclear accumulation of NFATc3 and NFATc2 in these cells. The broad TRPC blocker SKF-96365 inhibits these responses [39]. Therefore, TRPC1 plays a critical role in the regeneration process following muscle injury, by contributing to satellite cell activation.

A TRPC1 knockout (TRPC1^−/−^) mouse showed decreased endurance for physical activity. Histological analysis showed a reduced cross-sectional area of skeletal muscle fibers and myofibrillar protein content. Isolated muscle fibers from TRPC1^+/+^ mice showed instances of small, spontaneous activity which are absent in those from TRPC1^−/−^ mice. In primary muscle fibers, TRPC1 does not participate in store-operated or stretch-activated calcium influx. However, there is a marked reduction of force production in both the soleus and extensor digitorum longus (EDL) muscles of TRPC1^−/−^ mice. Furthermore, muscle fatigue is accelerated in the soleus and EDL muscles from TRPC1^−/−^ mice compared with those from TRPC1^+/+^ mice [88]. TRPC1-YFP transgenic mice also exhibited no significant differences in the electrical properties of skeletal muscle fibers. However, calcium clearance after repetitive contractile stimuli was delayed in TRPC1^−/−^ mice, and responses to cyclopiazonic acid were enhanced, suggesting that TRPC1 functions in the intracellular Ca^2+^ store membrane as a calcium leak channel (Fig. 2) [7]. In mdx mice, the diaphragm muscle had higher expression of TRPC1 compared with the sternomastoid and limb muscles. The levels of TRPC1 expression in mdx mice correlate well with the degree of pathological changes observed in skeletal muscles, i.e., the diaphragm shows the most severe pathological phenotype [43]. In a model of cardiotoxin-induced muscle injury, TRPC1^−/−^ mice showed significant hypotrophy and increased proportions of centrally nucleated muscle fibers. It is suggested that TRPC1^−/−^ myoblasts cannot correctly differentiate into myotubes because myogenic factors are downregulated. These phenotypes of TRPC1-depleted skeletal muscle were attributed to the suppression of the phosphatidylinositol-3-kinase-mammalian target of rapamycin (PI3K-mTOR) pathway. The TRPC1-mediated Ca^2+^ increase is critical for the activation of PI3K [89]. TRPC1^−/−^ muscle is resistant to repeated eccentric contraction. This phenotype is similar to that observed in muscle treated with streptomycin, a stretch-activated channel inhibitor. Although force reduction caused by repeated eccentric contraction was not affected by the absence of TRPC1, the loss of sarcolemmal proteins and reduced resting stiffness were suppressed by both TRPC1 knockout and streptomycin treatment, suggesting that TRPC1 contributes to stretch-activated Ca^2+^ entry in skeletal muscle [90].

The mechanical unloading seen in long-term bed rest patients and astronauts evokes muscle loss via oxidative stress. Ca^2+^ influx is critical for myoblast proliferation and controls exit from the G2/M phase of the cell cycle. Simulated microgravity, an in vitro model of mechanical unloading in space, reduced the expression of TRPC1 [6]. Hind limb unloading induces soleus muscle atrophy and reduction of tetanic force. During unloading, TRPC1 protein expression was reduced [84, 91] and recovered 14 days after reloading. The recovery of TRPC1 expression was preceded by and dependent on NFAT pathway activation. siRNA-mediated TRPC1 downregulation in vivo attenuated skeletal muscle regrowth of the soleus muscle, manifested by reduced cross-sectional area and type I myosin heavy chain expression [84]. These results suggest that proper mechanical signaling is important for skeletal muscle homeostasis, and TRPC1 plays a critical role in this.

Consistent with the accumulated data from the mdx mouse model, human myoblasts isolated from Duchenne muscular dystrophy (DMD) patients showed a significant increase in SOCE but no increase in levels of TRPC1, Stim1 or Orai1. However, pharmacological inhibition of phospholipase C or protein kinase C, which are components of a signaling complex with TRPC1, restores SOCE to the normal level [19].

Omega-3 fatty acid administration slows DMD progression, partly due to a reduction in TRPC1 expression [44].

Step up/down exercise involves concentric contraction in the right vastus lateralis (VL) muscle and eccentric contraction in the left VL muscle. Satellite cells in the left VL muscle only are activated, as indicated by an increase of expression of hepatocyte growth factor and MyoD, a myogenic transcription factor. As stated above, TRPC1 likely plays an important role in satellite cell activation. Consistent with this, TRPC1 expression was significantly increased in satellite cells of the left VL muscle, suggesting that eccentric but not concentric exercise activates satellite cells in a TRPC1-dependent manner [21].

## TRPC3

TRPC3 expression is relatively high in skeletal muscle tissue [32]. TRPC3 mRNA expression was increased after 3–4 days of differentiation in the C2C12 myoblast cell line [10, 40]. In the model of hind limb unloading, TRPC3 expression was lower in the early phase after the reloading process [91], suggesting that TRPC3 is downregulated during the regeneration process, possibly because undifferentiated myoblasts have lower levels of TRPC3 expression. TRPC3 channel expression in skeletal muscle is increased after neuromuscular activity by NFAT-dependent transcriptional upregulation. TRPC3 expression is higher in muscles enriched in slow oxidative fibers than those enriched in fast glycolytic fibers. Voluntary free-wheel running increased TRPC3 expression either 1 or 3 weeks after treatment in both the soleus and EDL muscles. Furthermore, electrical neurostimulation at 10 Hz increased levels of TRPC3 transcripts in the tibialis anterior (TA) muscle [66]. TRPC3 expression was significantly increased in TRPV4^−/−^ mouse skeletal muscle, in which the proportion of oxidative fibers was also increased [33]. These results suggest the importance of TRPC3 channels, especially in oxidative slow muscle fibers. TRPC3 interacts with ryanodine receptor type 1 (RyR1) in skeletal muscle (Fig. 2) [35, 80]. Loss of TRPC3 reduces the expression of RyR1, and vice versa [35], suggesting that TRPC3 plays a critical role in the modulation of RyR1. Indirect positive regulation of RyR by TRPC3 via Nox2-mediated ROS production has also been demonstrated in cardiomyocytes [29, 63, 64]. This TRPC3-Nox2-RyR coupling might also play important roles in skeletal muscle. TRPC3 also interacts with glucose transporter 4 (Glut-4) in T-tubules, and silencing of TRPC3 by siRNA reduced insulin-mediated glucose uptake by skeletal muscle. In accordance with these data, obese mice showed less oleoyl-acyl-sn-glycerol (OAG)-induced TRPC3 current [34]. TRPC3 also interacts with mitsugumin 29 (MG29), which is involved in the fatigue and aging processes of skeletal muscle. TRPC3-binding-deficient MG29 expression reduced the excitation-induced Ca^2+^ response in skeletal myotubes, indicating that MG29 plays a critical role in the regulation of TRPC3 channel function in skeletal muscle (Fig. 2) [83]. It has also been demonstrated that MG53 can interact with TRPC3 in skeletal muscle [1]. Myoblasts from muscular dysgenic mouse skeletal muscle failed to differentiate into myotubes when TRPC3 was knocked down [81].

TRPC3-overexpressing transgenic mice show a pathological phenotype similar to muscular dystrophy, suggesting that excess Ca^2+^ influx mediated by TRPC channels is sufficient to cause the disease. Using a TRPC6 dominant negative mutant, suppression of TRPC channels ameliorated the dystrophic myofibers of delta-sarcoglycan-null (Scgd^−/−^) mice [48].

## TRPC4

TRPC4 is also expressed in skeletal muscle cells, and its expression is increased in mdx mice. TRPC4 can form a heterotetramer with TRPC1. Similar to TRPC1, TRPC4 can interact with alpha-syntrophin and is part of the dystrophin-associated protein complex (DAPC) [67]. In human myoblasts, TRPC4 downregulation by siRNA or overexpression of a dominant negative mutant clearly suppressed SOCE, expression of the myogenic driver MEF2 and fusion of myoblasts into myotubes [2]. In these contexts, TRPC4 couples with TRPC1 and is regulated by STIM1L [3].

## TRPC6

TRPC6 expression is increased in mdx mouse skeletal muscle. Immunostaining revealed that TRPC6 is localized to the sarcoplasmic membrane [31]. Inhibition or deletion of TRPC6 has been reported to blunt the chronic mechanical stress-induced muscular contraction in mouse myocytes with Duchenne muscular dystrophy [68]. TRPC6 expression was significantly increased in TRPV4^−/−^ mouse skeletal muscle, in which the numbers of oxidative fibers were increased more than glycolytic fibers [33].

## Other TRPC channels

Compared with the aforementioned TRPC channels, the roles of TRPC2, TRPC5 and TRPC7 in striated muscles have been less well studied. The expression of TRPC2 is highly restricted, being present only in sperm and the vomeronasal sensory system [87]. Furthermore, TRPC2 is a pseudogene in the human genome. These facts imply that TRPC2 does not contribute significantly to striated muscle physiology. Although its specific function in striated muscles has not been demonstrated even with knockout mice, an involvement of TRPC5 in SOCE in cardiomyocytes has been implied. Recently, we demonstrated that extracellular ATP-induced Ca^2+^ influx mediated by TRPC5 induces nitric oxide synthase (NOS) activation and protects cardiomyocytes from hypertrophic responses [72]. TRPC7 was originally cloned from a cDNA library of mouse heart [56]. However, its function in cardiac and skeletal muscle remains elusive. The pathological significance of the closely related homologues TRPC3 and TRPC6 in striated muscles has been established, as mentioned above. Therefore, TRPC7 might play an important role in striated muscles, although confirmation of this will require a thorough analysis of knockout mice.

## Cardioprotective effect of exercise

Physical activity affects not only skeletal muscle cells but also other remote organs. Several factors secreted from skeletal muscle after exercise have been identified, and these are termed myokines [60]. However, not all effects of exercise have been reproduced by the administration of myokines, suggesting that the beneficial effect of exercise is not solely attributable to these limited factors but is a systematic change of whole tissues [28]. The heart is an example of an organ that is very sensitive to the effects of exercise [28]. Patients suffering from heart failure are recommended to engage in supervised physical activity to prevent disease progression and assist cardiac rehabilitation [5]. Therefore, a systematic understanding of the beneficial effects of exercise will be fundamental for developing more effective drugs against cardiac diseases.

## Physical exercise as a therapeutic intervention for DOX-induced cardiotoxicity

Doxorubicin (DOX) is a highly effective anticancer agent used to treat a variety of hematologic and solid malignancies [8, 79, 85, 92]. However, its dose-dependent cardiotoxicity limits its clinical use. The cardiotoxic effects of DOX range from asymptomatic increases in left ventricular (LV) wall stress to reductions in ejection fraction, arrhythmias and highly symptomatic congestive heart failure, which are all associated with high mortality [8, 14]. DOX initially causes the heart to shrink, which leads to induction of myocardial apoptosis and interstitial fibrosis at later stages of LV dilated cardiomyopathy [11, 94]. Numerous animal studies suggest that physical exercise training is the best intervention for preventing DOX-induced cardiac toxicity. In sedentary mice, DOX treatment resulted in a statistically significant decrease in heart function compared with control animals, which was mitigated by moderate aerobic exercise during DOX treatment. However, these protective effects of exercise were not observed when exercise was started after completion of DOX treatment. DOX caused not only a decrease in heart function but also cardiac atrophy and loss of body weight that were prevented by exercise, whereas non-trained mice exhibited no changes in these measurements. DOX delivery to the hearts of trained mice was reduced by consistent moderate aerobic exercise before DOX treatment [76]. Resistance training preserved cardiac function and attenuated the α- to β-myosin heavy chain shift that occurs with DOX treatment. No significant differences in lipid peroxidation were observed between sedentary and resistance-trained animals treated with DOX. [61]. Ashraf et al. demonstrated that after endurance training for 3 weeks, the DOX-induced increase in oxidative stress in the heart was significantly attenuated, possibly due to the upregulation of antioxidant systems such as superoxide dismutases (SOD) [4].

## TRPC3-Nox2 coupling in DOX-induced cardiac toxicity

**Fig. 3:**
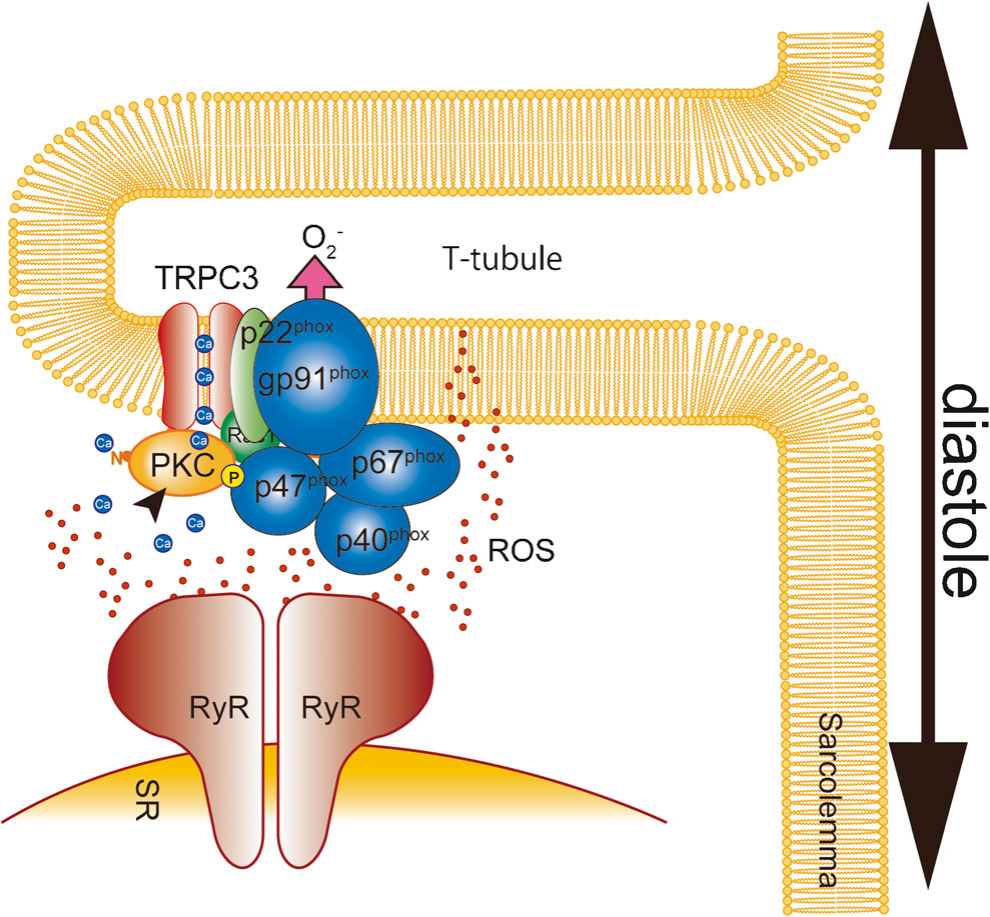
TRPC3-Nox2 coupling in cardiomyocytes. TRPC3 co-localizes and interacts with Nox2 in T-tubules in cardiomyocytes. Reactive oxygen species (ROS) oxidize ryanodine receptors (RyRs), which sensitizes them to Ca^2+^ during diastole

Nox2 proteins are known to play a critical role in DOX-induced cardiotoxicity. Indeed, Nox2 knockout (Nox2^−/−^) mice exhibit preserved contractile function after DOX treatment. In Nox2^−/−^ mice, DOX-induced structural changes of the heart including atrophy, interstitial fibrosis and apoptosis of cardiomyocytes were all diminished [93]. DOX treatment increased Nox2 expression in the heart [37, 69, 93]. Reduction of Nox2 expression by treatment with paeoniflorin ameliorated DOX-induced cardiomyocyte apoptosis [37]. We recently identified that TRPC3 is a critical regulator of Nox2 activity in the mechanically stressed heart [29]. TRPC3 functions in the signaling platform by providing Ca^2+^ to the downstream signaling molecules such as protein kinase C, which also plays a key role in Nox2 activation [51, 54]. TRPC3 knockout (TRPC3^−/−^) mice exhibit preserved cardiac function and heart mass compared with WT mice [69]. Furthermore, the DOX-induced increase of Nox2 protein abundance was significantly suppressed in the hearts of TRPC3^−/−^ mice. Although important for Nox2-mediated ROS production by providing Ca^2+^ for the enzymatic activation, TRPC3 may increase Nox2 protein abundance by preventing Nox2 from proteasome-mediated degradation through physical interaction (Fig. 3) [29]. Pharmacological inhibition or disruption of the TRPC3-Nox2 interaction ameliorates DOX-induced cardiac dysfunction [30, 69]. Therefore, suppression of TRPC3 function and/or expression will be promising therapeutic approaches against DOX-induced cardiac toxicity. Because TRPC3 expression is upregulated in various types of stressed hearts [9, 29, 53], the reduction of TRPC3 expression will lead to the reduction of ROS production by Nox2. Physical exercise reduced basal expression levels of TRPC3 and Nox2 in the mouse heart [69].

## Perspective

**Fig. 4:**
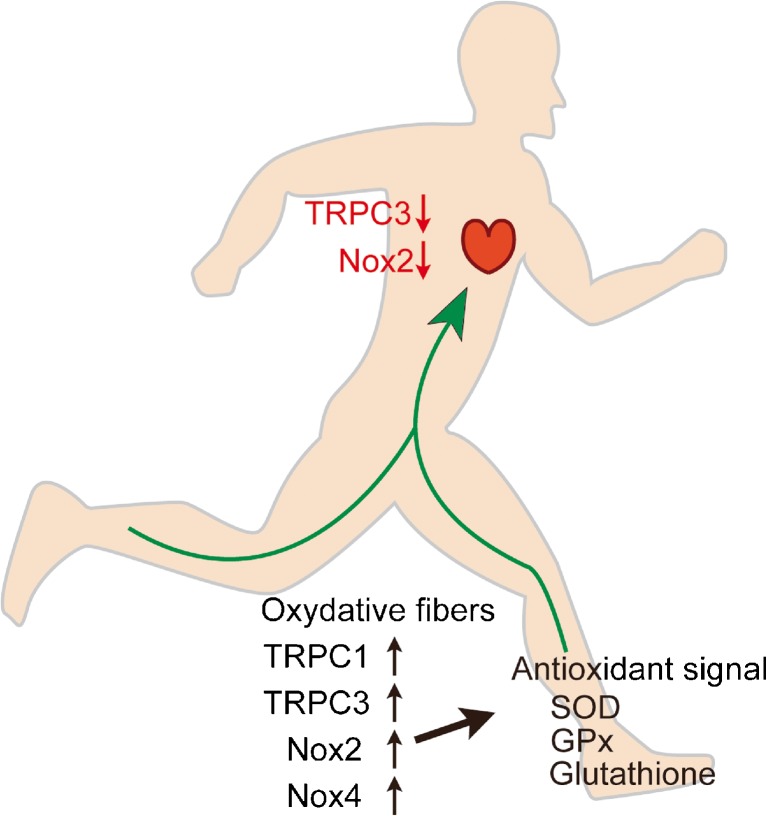
Physiological significance of canonical transient receptor potential (TRPC) channels in exercised human body. Exercise may increase the abundance of TRPCs and Nox proteins in skeletal muscle, while it may downregulate TRPC3 and Nox2 in the heart. Exercise-induced upregulation of TRPCs is concomitant with the upregulation of antioxidants, which may lead to a reduction of disease risk in remote organs, such as the cardiac pathological remodeling mediated by the TRPC3-Nox2 complex formation

The beneficial effects of physical activity for the prevention and improvement of several diseases are well accepted. However, the molecular details underlying those effects are still largely obscure. The most accepted concept is that exercise initially induces moderate ROS production that may be metabolized by promoting the endogenous antioxidant system [65, 73]. Because of an increase in endogenous antioxidants, the body becomes more resistant to the oxidative stresses which cause several pathological conditions. The Ca^2+^ influx induced by physical exercise plays an important role in mediating enhancement of muscular contractility and energy metabolism. As a signal mediator, Ca^2+^ also plays a critical role in the activation of Nox proteins. Increased activity of TRPC channels has been reported in skeletal muscle after physical activity [21, 66]. In skeletal muscle, acute exercise increases Nox2 protein activity. The increase of Nox2 activity and ROS is critical for the upregulation of manganese superoxide dismutase (MnSOD), glutathione peroxidase (GPx), citrate synthase (CS), mitochondrial transcription factor A (tfam) and interleukin-6 (IL-6) [20]. IL-6 is one of the myokines released by skeletal muscle during exercise, and its release is decreased by treatment with antioxidant [16, 86]. This evidence suggests that TRPC and Nox coupling is likely to be enhanced by physical exercise and contributes to the upregulation of adaptive responses against oxidative stresses in skeletal muscle. Furthermore, the increased activity of the antioxidative system in skeletal muscle is transduced to the whole body via secreted factors such as myokines to modify metabolic homeostasis (Fig. 4). In contrast, physical activity reduces Nox2 expression levels in heart, suggesting downregulation of the endogenous TRPC3-Nox2 protein complex (Fig. 4) [69]. Thus, the mechanical stress-induced upregulation of TRPC3 and Nox2 proteins is actually an important compensative mechanism to enhance Ca^2+^-dependent muscular contractility, and moderate exercise negatively regulates the formation of the TRPC3-Nox2 stable protein complex. It is clear that exercise-induced upregulation of TRPC3 and Nox2 is sufficient to upregulate endogenous antioxidant systems in skeletal muscles. However, it is unclear whether the formation of the TRPC3-Nox2 complex in skeletal muscles has the ability to enhance antioxidant systems. Recently, we have obtained the interesting finding that the upregulation of TRPC6 can suppress TRPC3-Nox2 functional coupling in hyperglycemic cardiomyocytes [55]. Although it has been widely accepted that TRPC6 forms a heterotetramer with TRPC3 and works cooperatively [58], the expression balance of TRPC channels might be flexibly changed and function to maintain homeostatic TRPC channel activity in a cellular context-dependent manner. Future studies focusing on the formation of the TRPC3-Nox2 complex in skeletal muscles will resolve the pathological significance of TRPC3-Nox2 protein-protein interaction in muscular organs, and we suggest that perturbation of the TRPC3-Nox2 complex may be an innovative strategy to imitate exercise-induced beneficial effects on cardiovascular systems.
